# Advancing sustainable RF energy harvesting for wearable electronics with 2.45 GHz textile-printed rectennas

**DOI:** 10.1038/s41598-025-09966-0

**Published:** 2025-07-08

**Authors:** J. Tavares, J. Lacik, P. Pinho, Z. Raida, H. Alves

**Affiliations:** 1 INESC MN, Instituto Superior Técnico, Lisbon, Portugal; 2 Brno University of Technoloy, Brno, Czech Republic; 3 Instituto de Telecomunicações, Universidade de Aveiro, Aveiro, Portugal

**Keywords:** Energy science and technology, Engineering, Materials science, Nanoscience and technology

## Abstract

The growth of IoT and wearable electronics demands sustainable energy solutions beyond short-lived, waste-generating batteries. RF energy harvesting offers a self-powered alternative by capturing ambient RF energy. However, implementing this technology on textile substrates remains challenging due to material incompatibility, ink toxicity, substrate porosity, and scalability constraints. This study addresses these challenges by developing optimized fabrication techniques for printed textile rectennas operating at 2.45 GHz. It focuses on conductive ink formulations tailored for textiles, scalable integration methods such as screen-printing and doctor blade techniques, and improved attachment methods for lumped components, ensuring full integration of a microstrip patch antenna and rectifier circuit onto fabric. The research systematically examines the impact of substrate porosity, ink adhesion, material losses, mechanical deformation, dielectric variability, and surface roughness on energy harvesting efficiency. Additionally, it promotes environmentally sustainable solutions by reducing reliance on volatile organic compounds (VOCs) and complex fabrication processes. Electromagnetic simulations and experimental validations confirm the rectenna’s capability to harvest 2.4 GHz ISM band energy, despite challenges such as dielectric sensitivity and conductive ink losses. This work establishes a scalable, cost-effective framework for next-generation wearable and IoT applications, advancing flexible electronics and self-sustaining smart textiles.

## Introduction

Internet-of-things (IoT) networks predominantly rely on sensors powered either by cables or batteries. In wearable electronics, cables are impractical, while batteries are limited by their short operational lifespans. Frequent battery replacements, not only exacerbate supply challenges but also contribute significantly to electronic waste. This accentuates the urgent need for sustainable, portable, and flexible power sources that can address these limitations effectively^1^. Radiofrequency (RF) energy harvesting offers a promising solution for powering electronic devices sustainably. While efficient RF energy harvesters have been successfully developed on rigid substrates like silicon and III-V semiconductor compounds, scaling this technology to large-area, flexible electronics systems remain a significant challenge, particularly for wearable substrates such as textiles. Recent advancements have led to the developments of textile rectifiers and rectennas, aligning with the vision for IoT integration. However, these devices face several critical challenges, with one major issue being material mismatches. Particularly, antennas are typically fabricated using textile dielectrics, while rectifiers often rely on plastic dielectrics. To address this, rigid substrates are sometimes adhered to textile surfaces, but this approach often intensifies incompatibility problems, resulting in pronounced performance limitations. In efforts to create fully textile rectennas, compact and stable textile dielectrics like felt have been employed. These devices often incorporate conductive fabrics, metallic foils, or polymeric laminates, which are attached to the textile dielectric through techniques like lamination or sewing (Table 1). However, these attachment methods introduce significant challenges, including high costs, increased fabrication complexity, and limited scalability. The production processes, often relying on labor-intensive and expensive techniques such as photolithography, sewing and lamination, also add weight and compromise mechanical flexibility, negatively affecting device performance and wearability^2–6^.

Attempts to print rectennas directly onto textiles have faced significant challenges due to the material’s inherent porosity and roughness^7,8^. Additional complications arise from the conductive inks, which are typically fluid formulations based on organic solvents such as ethylene glycol and butyl carbitol. These solvents not only pose significant health and environmental risks due to their toxicity but can also adversely affect the properties of the textiles, including color, hydrophilicity, and texture. Furthermore, these inks are primarily designed for use substrates such as paper, or plastic, lacking the flexibility, fiber penetration and adhesion, wash resistance, and durability required for porous, wearable, and stretchable materials^9–11^. Some efforts have explored the use of printing techniques on fabric with conductive inks containing volatile organic compounds (VOCs)^12–15^. In one example, it was achieved a suitable output with an array of rectennas, resorting to a Metallized ink^15^. However, this approach required high curing temperature (375 ºC) and contains butyl carbitol (VOC), which can adversely affect textile substrates and compromise durability. Furthermore, the environmental risks associate with VOCs remain significant^12–16^.

Attachment methods for the lumped components, such as the use of epoxies, introduce additional complications. For example, epoxies often require long curing times, are brittle under variable conditions, and add to the overall cost and fabrication complexity^17^. These factors introduce additional challenges for the development of durable and cost-effective textile-based rectennas. Some promising results were achieved using antenna arrays, though these outcomes can be partially attributed to the advantages of the rectenna array itself rather than solely to the properties of the ink^15^. This highlights the need for further innovation in ink formulations, attachment methods, and processing techniques adapted specifically for textile-based rectennas. Table 1 provides a summarized comparison of flexible rectennas from the state of the art, emphasizing key performance metrics, fabrication techniques, and material choices.

**Table 1:** Comparison of flexible rectennas from state of the art.^20–22^

Substrate(s)	Dielectric permittivity	Conductive material	Fabrication method	Working Frequency (GHz)	Input Power (dBm)	Refs.
Kapton	3.4	Silver ink	Direct-write dispenser printing	413 and 915 MHz	6	^18^
Kapton	-	MoS_2_, Pd, Ti, Au	Electron-beam evaporation and electron-beam lithography	8–12 GHz	-15–5	^19^
PDMS, Flexible PCB	2.5, -	Ag-based conductive ink, Eutectic Gallium– Indium liquid metal and Copper	Desktop photo inkjet printer and coating	2.45 GHz	0–30	^20–22^
PDMS	2.7	Flexible graphene macroscopic films	Laser direct molding engraving	2.45 GHz	-15–10	^23^
Cotton canvas	1.67	Conductive fabric (tungsten-copper- nickel alloy and polyester fiber)	Metallic patterns cutting, transfer and thermal releasing	2.45 GHz	-20–10	^24^
Roger 3003	3	-	-	24 GHz	0–20	^25^
Textile woven Polyester and Flex PCB	1.67	Copper laminates	Photolithography	24 GHz	20	^26^
Weft-knitted fabric	1.385	Pure Copper Taffeta Fabric	Lamination	5.8 GHz	-15–15	^27^
Fabric	2.75	Conductive thread	Embroidery	2.45 GHz	-20–8	^28^
Cotton Fabric bandage and Kapton	1.2, -	Silk-coated copper Litz wire	Embroidery, -	915 MHz	-10–10	^29^
Polyester threads	1.8	Polyamide with silver coating	Woven technology	2.4 GHz	-10	^30^
Cotton fabric	1.6	Conductive yarn	Digital Embroidery	2.45 GHz	-30–20	^31^
Cotton tee-shirt	-	Metallized ink (NovaCentrix Metalon HPS-FG57B)	Screen-printing	2–5 GHz	-	^15^
Cordura textile fabric	1.93	Copper Foil	-	2.45 GHz	-40–0	^32^
Jeans cotton	1.7	Copper foil tape	Foil tape	2.45 and 5.8 GHz	-20–15	^33^

Textile-based rectennas face additional challenges, including material incompatibility, ink toxicity, substrate porosity, and constraints in scalability. Fabrics like cotton, which absorb moisture, have dielectric properties sensitive to humidity, causing shifts in resonant frequency and reducing energy efficiency. While textile flexibility offers advantages for wearables, it also introduces electrical inconsistencies, making standardized performance comparisons difficult across designs^2^. Furthermore, mechanical deformation, surface roughness, and frequency detuning during fabrication reduce energy harvesting efficiency, especially when compared to the stable performance of silicon-based systems. These challenges highlight the need for innovative strategies to enhance both reliability and scalability, requiring specialized fabrication methods that account for the unique properties of textiles.

This study aims to address critical challenges in the development of wearable RF energy harvesters, including material incompatibility between textile antennas and rectifiers, the lack of environmentally friendly and scalable fabrication methods suitable for porous textiles, and the mechanical and electrical limitations introduced by conventional component attachment techniques. By systematically analyzing how material properties and fabrication processes influence device performance, this work develops and validates scalable, cost-effective, and sustainable techniques for printing textile-based rectennas. The primary objective is to demonstrate the feasibility of fully integrating both antenna and rectifier directly onto flexible textile substrates using improved conductive inks and low-temperature fabrication processes. Experimental evaluation under realistic mechanical and RF conditions provides new insights into the performance, durability, and potential of next-generation sustainable textile-based RF energy harvesters.

## Results and discussion

A microstrip patch antenna was selected as the receiving antenna for the textile rectenna due to its simplicity, versatility and suitability for analyzing factors contributing to energy losses and exploring strategies to mitigate them. This antenna design, composed of a dielectric substrate sandwiched between a conductive radiating patch and a ground plane, is particularly well-suited for understanding the factors affecting performance. Due to its simple and well-defined structure, the microstrip patch antenna is well-suited for studying performance-affecting factors, as its design parameters – such as patch dimensions, substrate properties, and material losses – can be systematically analysed and optimized to evaluate energy losses and enhance efficiency^34,35^. In this study, the dielectric material selected was Sintex 3D097 textile, a flexible substrate with a thickness of 2.6 mm and a low relative permittivity (ɛ_r_) of 1.22. Its low loss characteristics, evidenced by a loss tangent of tan δ ≈ 0.001, made it particularly adequate. Prior work^17^ using similar substrates and planarization methods demonstrated that these textile structures maintain stable RF performance under mechanical deformation, supporting the expected reliability of the current approach. However, the material’s physical properties -such as flexibility and porosity, which contribute to high surface roughness- introduce challenges during fabrication^17,36^.

To address these issues, a conductive ink with a high conductivity was developed to reduce resistive losses and ensure efficient current flow in both the patch and ground plane layers. Additionally, the ink was engineered to exhibit excellent adhesion after deposition to minimize scattering losses and ensure consistent electromagnetic performance. Three different inks (ink **a**, **b** and **c**) were formulated, all incorporating silver nanoparticles (Ag NPs) to ensure high electrical conductivity. Each ink differed in its choice of solvent and binders, tailored to enhance performance with an appropriate viscosity and conductivity. The viscosity and the solid/liquid content ratio of the ink are crucial to ensuring the adhesion of the ink to the substrate, with different needs arising when printing on different substrates^7,8^. Ink **a** matrix was prepared using a nonconductive fluorelastomer (DAL-EL LT 302, Daikin Chemical Europe GmbH) dissolved in acetone. The acetone served to dissolve the elastomer enabling to effectively combined with silver nanoparticles. The proportion used ensured a proper viscosity between 16 000 and 24 000 mPas, adequate for screen-printing and doctor blade processes. The conductive ink provided good electrical performance for antennas and circuit design, whereas the printed film has an electrical conductivity of 15 091 S∙m^− 1^. Ink **b** used a water-based adhesive as matrix and Poly(3,4-ethylenedioxythiophene) polystyrene sulfonate (PEDOT: PSS) in addition to the AgNPs. PEDOT: PSS is a conductive polymer known for its low loss properties, biodegradability, recyclability, and hydrophobic properties^7,8,37^. However, its electrical conductivity is low, 1594 S∙m^− 1^, lower than inks **a** and **c**, not being suitable for rectifier circuits devices that require high electrical conductivity across all printed lines. This limitation is likely due to the fabrication process used, which may not be optimal for this ink. Nevertheless, additional studies were conducted with this ink to gain a better understanding of the material’s impact. Ink **c** consists of Ag NPs, a water-based adhesive and water, prepared as described previously^17^. Ink **c** is an eco-friendly and sustainable ink, and it was developed to be applied for screen- and transfer- printing processes. Its electrical conductivity is similar to ink **a**, with an electrical conductivity of 14 179 S∙m^− 1^. To evaluate the performance of these three inks, microstrip lines were designed and fabricated to study their associated losses and suitability for the textile substrate.

To align with the objective of studying these devices through scalable printed methods, the work focused on employing screen-printing and doctor blade techniques. While the two methods are similar, their processing differences often result in variations of the coated films. In screen printing, factors such as mesh size, squeegee pressure, and ink viscosity can influence the thickness of the printed layer. In doctor blade, the layer thickness is determined by the angle of the blade, pressure and ink viscosity. These factors can lead to variations in both thickness and uniformity, which may increase electrical resistance. The viscosities of the three inks (ink **a**, **b**, and **c**) all fell within the 16 000–24 000 mPas for both techniques, ensuring that uniform layers could be achieved without significant trade-offs in conductivity or adhesion across the printed layers. Nevertheless, both methods are simple, low-cost, scalable, and employ widely used industrial textile processes for patterning. This demonstrates that screen-printing and doctor blade methods can be effectively adapted for flexible textile substrates. Notably, specific formulations, such as ink **a**, enable strong adhesion due to their optimized viscosity, while also producing low-resistance conductive layers, both of which are critical for energy-harvesting applications^7,8^.

**Fig. 1:**
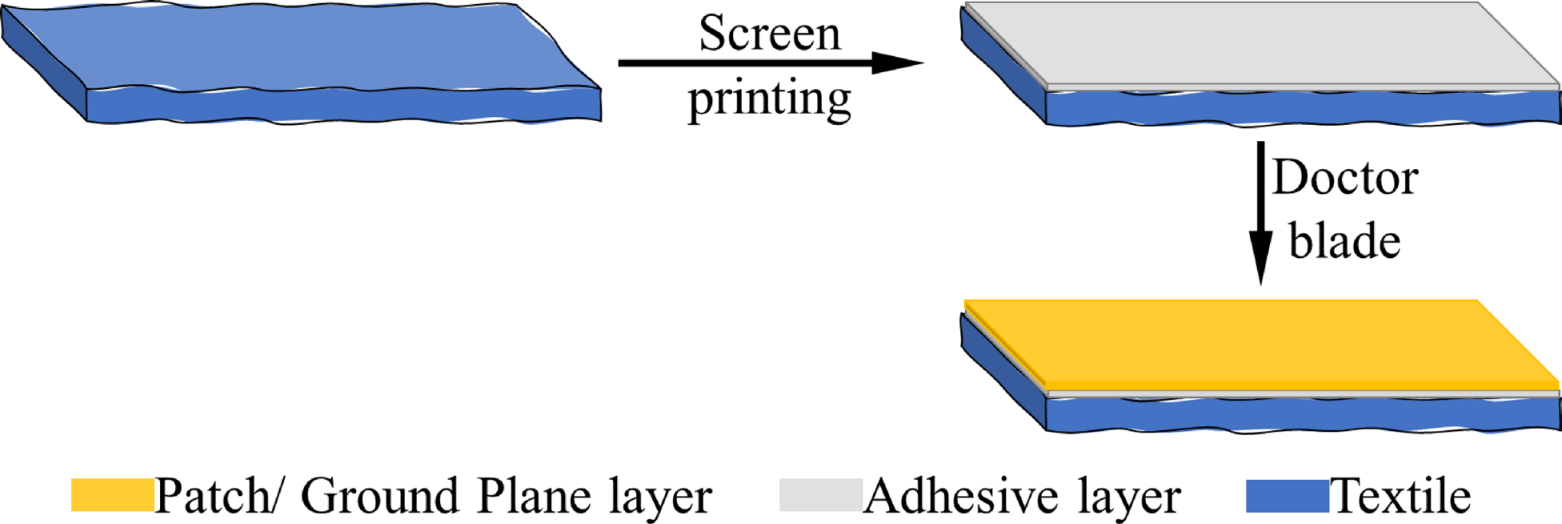
Fabrication process of the RF components.

Figure 1 illustrates the fabrication process for the RF components (e.g. antennas, microstrip lines, rectifier circuits). The first step involves screen-printing a water-based adhesive onto the textile surface to ensure planarization, using a screen mesh with a pore size of 133 μm. This pore size mesh allows the adhesive particles to penetrate adequately, uniformly covering the textile’s dielectric surface while maintaining its flexibility. Given the textile´s porous and rough surface, the adhesive layer is essential to prevent the printed conductive film from breaking and creating discontinuities. Without this layer, the conductive film would conform to the textile’s roughness, resulting in a non-planar surface and increased electrical losses^38^. Additionally, the planarization prevents the conductive film from leaking into the porous textile, thus avoiding short circuits. After allowing the adhesive layer to dry for 30 min at room temperature, the conductive layer was deposited onto the planarized textile surface using the doctor blade method. Doctor blade processing was chosen over screen printing due to the particle size of the AgNPs (approximately 1–3 μm) and the size of some ink binders, which were too large for standard screen printing meshes. This conductive layer was then left to dry at room temperature for 30 min. The same process was repeated for the three inks for each component.

To enhance the device performance, energy losses were analyzed, focusing on conductor, dielectric, and radiation losses, which are influenced by the materials and fabrication processes. Microstrip lines are subject to three primary types of energy losses: conductor, dielectric, and radiation losses^39^. Conductor losses occur due to resistivity of the conductive material, which is influenced by factors such as conductivity, surface roughness, and thickness relative to the skin depth. Dielectric losses arise from the substrate material’s inherent properties, such as permittivity and loss tangent. Substrate with lower dielectric constants generally exhibit reduced dielectric losses, but may lead to increased radiation losses. Electromagnetic losses are influenced by geometry of the circuit, conductive material and the substrate’s thickness and composition^40^. In textiles, dielectric losses tend to remain minimal due to their low permittivity values, as in the Sintex 3D097 textile. As the dielectric constant value is lower, the concentration of the electromagnetic energy in the substrate decreases, and radiation losses increase^41^.

**Fig. 2:**
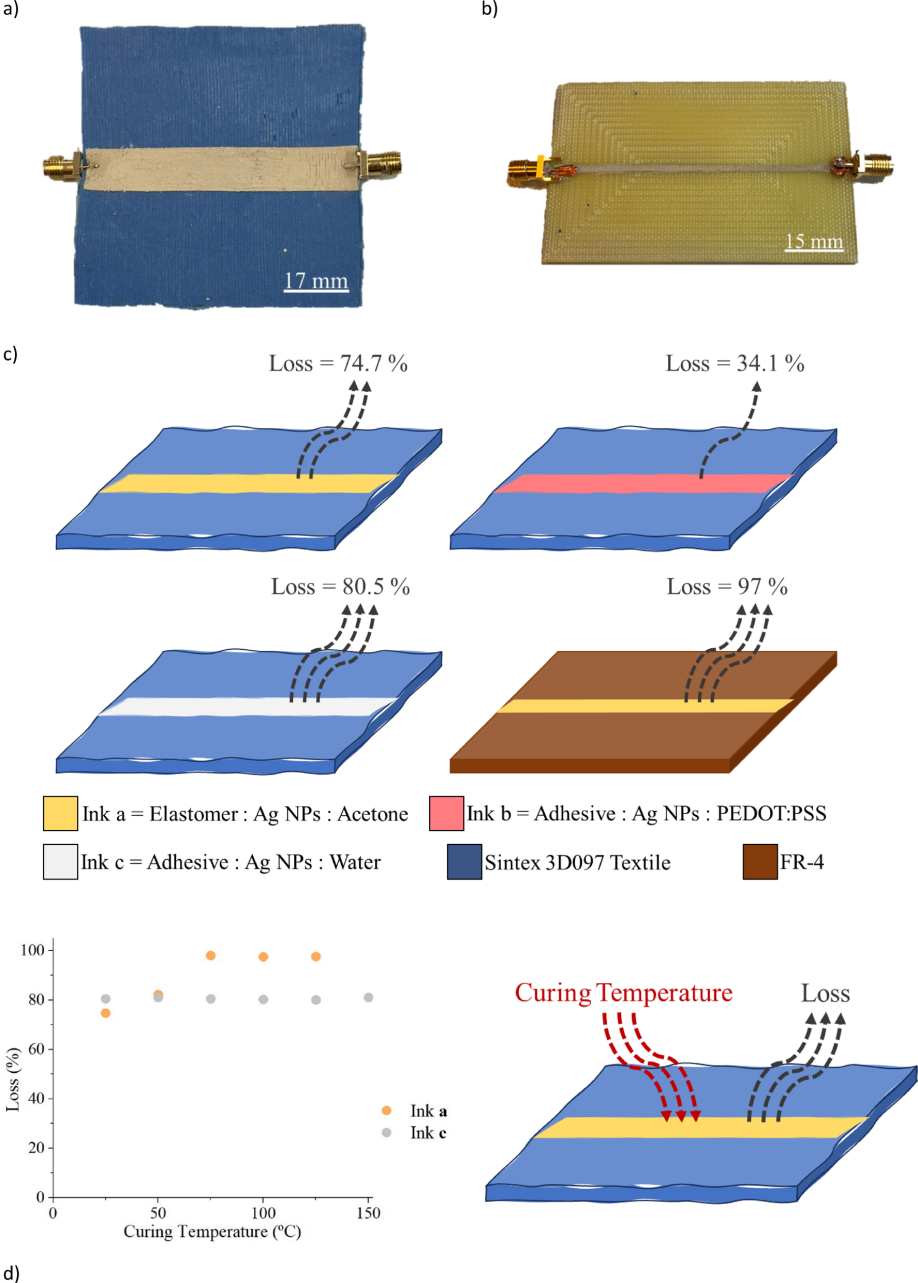
(**a**,** b**) Photographs of 50 Ω microstrip lines fabricated on (**a**) the Sintex 3D097 textile substrate and (**b**) a standard FR-4 dielectric substrate, using the design parameters simulated in CST Studio Suite. (**c**) Electromagnetic loss measurements of microstrip lines printed with inks a, b, and c on both dielectric substrates, highlighting the influence of ink composition and substrate material on conductor and dielectric losses. (**d**) Impact of curing temperature on the losses of microstrip lines fabricated with inks a and c, showing the thermal sensitivity of ink a, which exhibited increasing losses with temperature due to stiffening of the fluorine rubber matrix. Losses were quantified through S-parameter measurements using a PNA-X Network Analyzer.

To investigate the impact of conductive ink formulation and dielectric substrate properties on electromagnetic losses, 50 Ω microstrip lines were designed using CST Studio Suite and fabricated on two substrates: a conventional FR-4 laminate and the Sintex 3D097 textile. The microstrip line configuration consisted of a conductor trace on one side of the dielectric and a ground plane on the opposite side (Fig. 2a, b). The geometry was tailored to maintain a characteristic impedance of 50 Ω, based on the dielectric constant of each material. For Sintex 3D097 (ε_r_ ≈ 1.7), the resulting line width was 11.28 mm and length approximately 70 mm; for FR-4 (ε_r_ ≈ 4.5), the width was 2.8 mm and length ~ 84 mm. The width is primarily determined by the dielectric constant to ensure impedance matching, while the line length affects phase characteristics but not impedance directly.

Each set of microstrip lines was fabricated using the three conductive inks **(a**, **b**, and **c**), following the process outlined in Fig. 1. After printing and curing, S-parameter measurements were conducted using a Keysight PNA-X Microwave Network Analyzer to quantify conductor and dielectric losses.

Figure 2c presents the electromagnetic loss measurements for each ink on both FR-4 and textile substrates. Among the three inks, ink **b** demonstrated the lowest losses (~ 34.1%), owing to the inclusion of PEDOT: PSS – a polymer with inherently low dielectric loss. However, its low DC conductivity (~ 1594 S/m) made it unsuitable for radiating elements, and it was excluded from further studies. Of the remaining inks, ink **a** exhibited lower conductor losses (~ 74.7%) than ink **c** (~ 80.5%). This difference is attributed to ink **a**’s optimized solvent and binder system, which better preserved the conductive network during curing. These findings were supported by simulations, which confirmed that in the textile-based lines, the total losses were primarily due to conductor characteristics, as the textile substrate (tan δ ≈ 0.001) exhibited substantially lower dielectric loss than FR-4 (tan δ ≈ 0.03).

To assess thermal stability, additional samples using inks **a** and **c** were cured at temperatures ranging from room temperature to 150 °C in 25 °C intervals. The results, shown in Fig. 2d, revealed that ink **c** maintained stable performance across the entire temperature range, while ink **a** showed a marked increase in losses with higher curing temperatures—from 74.7% at 25 °C to 97.6% at 125 °C, with complete failure at 150 °C. This degradation is linked to the fluorine rubber matrix in ink a, which offers flexibility at low temperatures but becomes brittle at elevated temperatures, disrupting the conductive path.

Based on these findings, ink **a** was selected for further device development, using room-temperature curing to preserve both electrical performance and mechanical flexibility. This choice balances process scalability, RF performance, and substrate compatibility.

Compared to previous studies that used PEDOT: PSS-based inks – which, while low-loss, lack sufficient conductivity for radiating structures – or brittle oxide inks, this work demonstrates that a carefully formulated ink–substrate combination, supported by proper planarization, can yield low-loss, flexible, and scalable RF structures suitable for wearable applications.

To complement the results shown in Fig. 2; Table 2 provides a concise summary of the most relevant quantitative parameters for the evaluated conductive ink formulations. This includes electrical conductivity, RF losses at 2.45 GHz, and curing condition, which collectively informed the selection of the optimal ink for subsequent device fabrication.

**Table 2:** Electrical conductivity and RF loss of the tested inks at 2.45 GHz.

Ink	Conductivity (S/m)	RF Loss (%) @2.45 GHz	Curing Condition	Evaluation
Ink a	~ 15 091	~ 74.7	Room Temperature	Suitable
Ink b	~ 1 594	~ 34.1	Room Temperature	Low conductivity
Ink c	~ 14 803	~ 80.5	Room Temperature	High RF loss

As summarized above, while ink **b** offered the lowest RF loss, its low DC conductivity made it unsuitable for rectifier interconnections or radiating elements. Ink **c**, though comparable in conductivity to ink **a**, exhibited higher conductor losses. Ink **a** was ultimately selected due to its favorable balance of electrical conductivity, RF performance, and compatibility with low-temperature processing on flexible substrates. All subsequent antenna, rectifier and rectenna measurements presented in this work were performed using ink **a**.

**Fig. 3:**
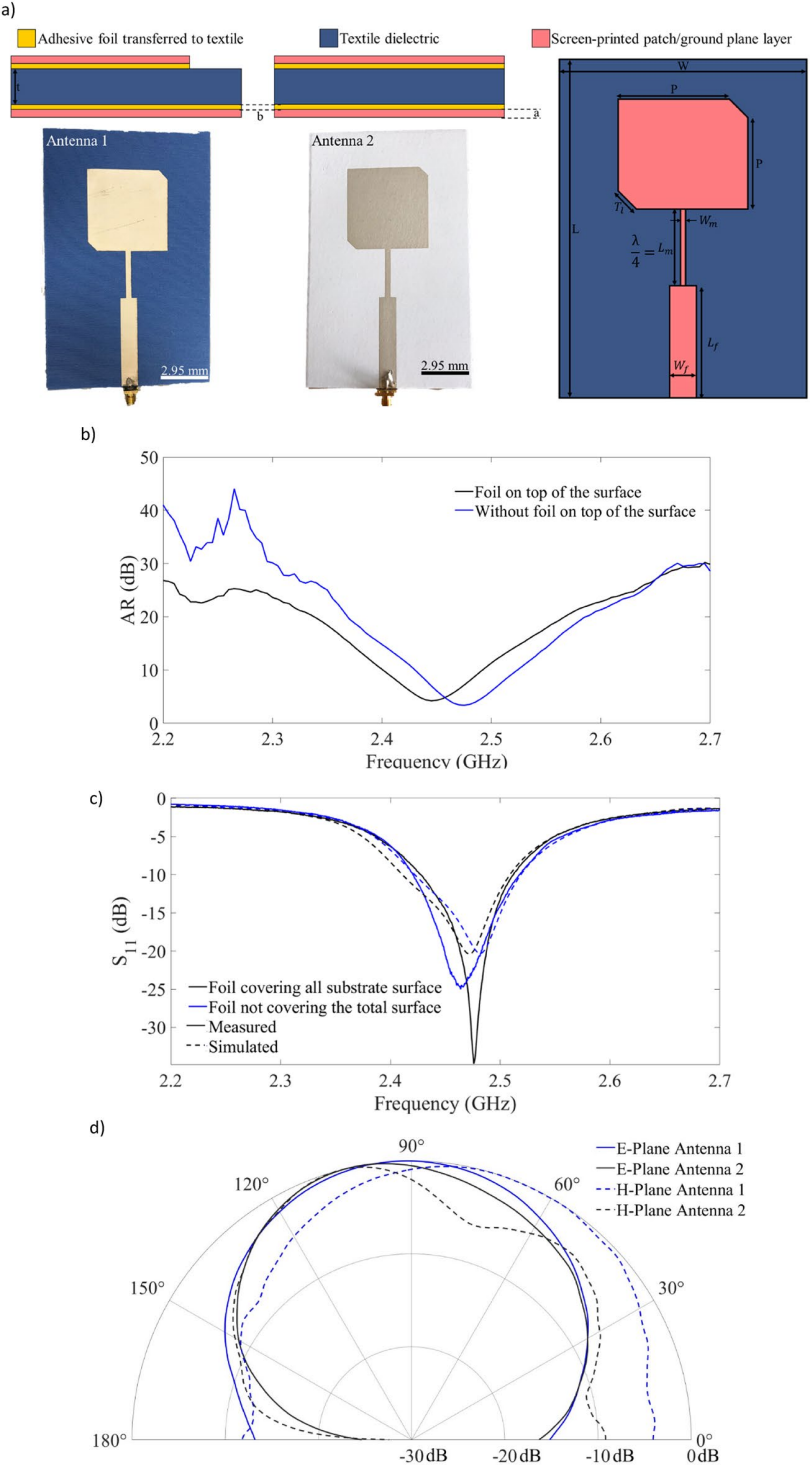
(**a**) Antennas **1** and **2** were designed with distinct geometries: Antenna **1** lacks adhesive foil covering the entire surface, while and Antenna **2** features adhesive foil covering the entire dielectric substrate. The geometry design, including surface and cross-section views, is illustrated alongside corresponding photography for each cross-section case. (**b**) Axial ratio of the antenna: circular polarization. (**c**) Reflection coefficient response simulation and measurements to each antenna, and (**d**) comparison of the measured radiation patterns for both antennas.

To address energy harvesting applications, a straightforward circularly polarized antenna design was developed to maximize energy capture from all directions, utilizing the textile as the dielectric and ink **a** as the conductive element. The design incorporated a microstrip patch antenna and the associated $\:\frac{\lambda\:}{4}$ matching line optimized for the center frequency of 2.4 GHz ISM band, calculated using the standard microstrip patch antenna design equations^34^. These equations were used to determine the optimal dimensions and geometry for the antenna. The resulting design, including both the surface layout and cross-sectional view, is illustrated in Fig. 3a. The dimensions for each parameter are detailed in Table 3. Here, **W** and **L** represent the width and length of the antenna, while **P** denotes the side length of patch. **W**_**f**_ and **W**_**m**_ are widths of the feed and matching lines, respectively, and **L**_**f**_ and **L**_**m**_ correspond to their lengths. The matching line was design to be a quarter-wavelength ($\:\frac{\lambda\:}{4}$). Lastly, **T**_**l**_ defines the antenna’s polarization and was adjusted to achieve the desired circular polarization, resulting in a low axial ratio (Fig. 3b).

**Table 3:** Physical dimensions of the antenna design.

Antenna design geometry	Dimensions (mm)
W	100.1
L	158.1
P	50.1
W_f_	10.7
L_f_	28.9
W_m_	3.7
L_m_	29.1
T_l_	5.6

For the antenna design, two options were evaluated: one with adhesive foil applied only beneath the patch (Antenna **1**) and another with an adhesive foil covering the entire surface (Antenna **2**). The adhesive foil ensures the planarization of the textile surface, enhancing the precision of the printing processes and minimizing material losses but can influence the antenna performance. Simulation using both options (**1** and **2**) were performed using CST Studio Suite, evaluating scenarios where the adhesive foil covered the entire surface and where it was applied solely beneath the patch. Both configurations were fabricated, and their reflection coefficient measurements were compared with simulations results. Figure 3a presents the cross-section designs of the two configurations, accompanied by corresponding photographs of the antennas. For Antenna **1**, simulations predicted a resonant frequency of 2.48 GHz with an S_11_ value of -20.3 dB, while measurements showed a slightly shifted resonant frequency of 2.46 GHz with an improved S_11_ value of -25 dB. Similarly, for Antenna **2**, simulations anticipated a resonant frequency of 2.47 GHz with an S_11_ signal of -20.4 dB, but measurements revealed a resonant frequency of 2.48 GHz with a significantly enhanced S_11_ value of -34.8 dB (Fig. 3c). Despite these minor discrepancies, both antennas operate effectively within the 2.4 ISM band frequency range. Studies suggest that full adhesive coverage can influence the dielectric permittivity of the textile substrate, as it alters the material porosity^17^. Textile are composed of small, uniformly distributed pores, filled with air, that contribute to their overall dielectric properties. When adhesive fully covers the textile (as in Antenna **2**), these pores are sealed, reducing the air content within the material and impacting the dielectric constant. CST Studio Suite simulations do not account for the intrinsic porosity of textile substrates, which contributes to the observed discrepancies between simulated and measured resonant frequencies. To address this limitation, this study adopts an empirical approach to analyze the effects of substrate porosity, dielectric variability, and ink adhesion on device performance. By systematically comparing antenna and rectifier configurations with different adhesive coverages and ink formulations, we infer the role of these material and process parameters in influencing electrical behavior, particularly in terms of impedance matching, reflection losses, and rectification efficiency. Additionally, Antenna **2**, with full adhesive coverage, exhibited a narrower reflection coefficient bandwidth. This can be attributed to the adhesive providing a completely planar surface, which improves the accuracy of the printing process and enhances the connection with the SMA connector. However, full coverage reduces the flexibility of the fabric, presenting a trade-off between performance and mechanical properties. Despite these differences, both antennas demonstrated effective operation within the target frequency, highlighting the practical feasibility of both designs.

In addition to the reflection coefficient measurements, the radiation pattern of both antennas was also measured, with the results presented in Fig. 3d. These measurements were conducted in an anechoic chamber. As observed, the radiation patterns of the two right-hand circular polarized (RHCP) antennas exhibit slight differences. Antenna **1** shoes a broader half-power beamwidth and a more circular radiation pattern, while Antenna **2** demonstrates a slightly more directive radiation pattern. Based on these observations, the design with adhesive applied only beneath the patch (Antenna **1**) was selected for further developments, to enhance the energy harvesting capability.

To enable energy harvesting with the fabricated and characterized antenna, two key functional blocks were designed and integrated into a single circuit: a matching network and a rectifier circuit. The matching network ensures a 50 Ω impedance match between the antenna and the rectifier circuit, minimizing losses from impedance mismatch. The rectifier circuit converts the RF signals harvested by the antenna into usable DC voltage, providing power for devices such as sensors.

In designing the rectifier circuit, a single shunt diode rectifier was selected from existing rectifier configurations due to its simple structure, high conversion efficiency, and compatibility with the precision requirements of screen-printing processes. This design also enables the creation of a continuous pattern. Schottky diodes were chosen for their low forward voltage and high conversion efficiency, which simplifies impedance matching. Among available options, the SMS7630 diode, commonly used for RF energy harvesting in the 2.4 ISM band was utilized^42^.

**Fig. 4:**
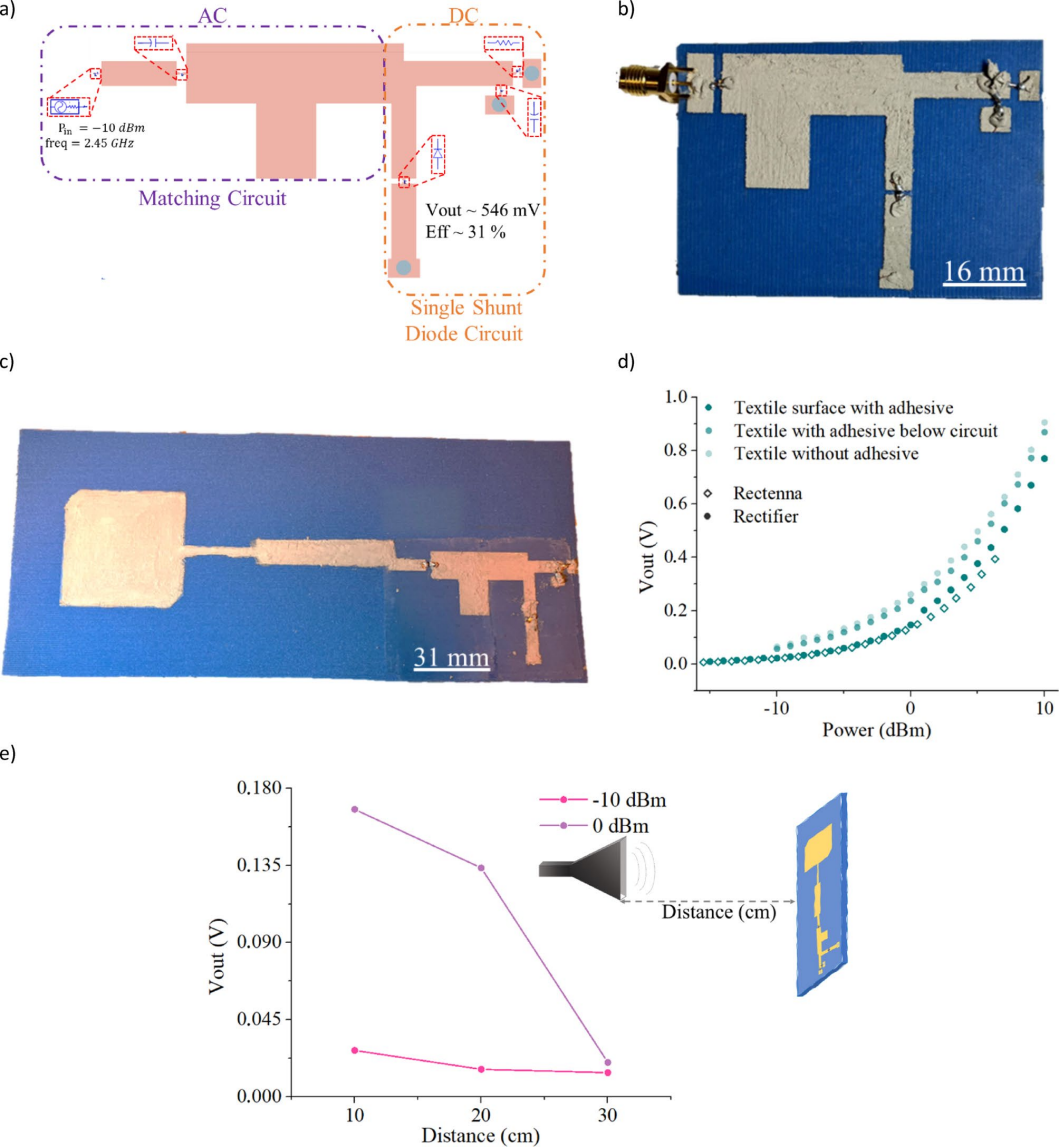
(**a**) Rectifier circuit design with its simulated efficiency and outpout voltage at -10 dBm; Textile (**b**) rectifier and (**c**) Rectenna; (**d**) Output comparison between textile rectenna and textile rectifiers (with adhesive over the textile surface, adhesive below the circuit and without any adhesive over the textile dielectric); and (**e**) Rectenna output voltage with different distances at -10 and 0 dBm of input powers.

The circuit design, consisting of a matching network and a single shunt diode rectifier (Fig. 4a), was developed using Advanced Design System 2022 (ADS) software. The design employs the same materials as the antenna, with textile as the dielectric substrate and conductive ink **a** for the circuit traces. Within the matching network, a stub and a series capacitor acted as an input filter, reducing signal reflection and enhancing RF-to-DC conversion efficiency. A shunt capacitor placed after the diode smoothed the DC output waveforms. Additionally, a quarter-wavelength ($\:\frac{\lambda\:}{4}$) transmission line was placed between the stub and the diode to ensure the circuit operated as a full-wave rectifier, rather than a clamping circuit^34^. The final design yielded a simulated DC output voltage of approximately 546 mV and an efficiency of ~ 31%.

Similar to the antennas, different textile rectifiers were fabricated with the same design but varying adhesive deposition: one with adhesive covering the entire textile surface (Fig. 4b), another with adhesive applied only beneath the conductive lines, and one without any adhesive layer between the textile and the conductive lines. The fabrication process consistently followed the steps outlined in Fig. 1. The lumped components were placed and connected with the conductive ink **a**. The conductive ink **a** with its viscosity allowed a great fixation of the SMD components. After the fabrication, each rectifier circuit was measured, and their output voltage was recorded for different input power levels (Fig. 4d). For the rectifier circuit without adhesive and with adhesive only below the circuit lines, the behavior is similar. When increasing the input power from − 10 to 10 dBm, the output voltages goes from 64.8 mV to 0.91 V and from 57 mV to 0.87 V, respectively. For the rectifier with the textile surface all planarized with adhesive, when increasing the input power from − 15 to 10 dBm the output volage increases from 9.4 mV to 0.77 V. The adhesive slightly reduced the output, which is expected as the textile’s dielectric constant is altered due to the covering of its pores. However, these differences were minimal. Therefore, to simplify the printing process, the adhesive was applied to cover the entire surface of the rectifier when building the rectenna (Fig. 4c).

The rectenna was fabricated by printing the rectifier circuit and the antenna directly onto the same substrate, maintaining full flexibility and eliminating the need for rigid connection such as SMA connectors. While the fabrication process remained consistent with that used for standalone components, the new design integrated the antenna and rectifier through impedance matching lines, each designed with a 50 Ω characteristic impedance to ensure proper matching. This fully printed and integrated structure reflects a fabrication strategy specifically selected to balance mechanical flexibility and electrical functionality. It involved the use of textile-compatible conductive inks, scalable deposition techniques such as screen-printing and doctor blade coating, and a direct ink-based attachment method for lumped components, enabling the realization of a seamless, textile-based RF energy harvester. To evaluate the system’s performance, the output voltage of the rectenna was measured for different input power levels and compared to that of the standalone rectifier with adhesive backing. As expected, the rectenna’s output closely resembled the behavior of the rectifier tested under similar surface coverage. For input power levels ranging from − 15 to 6 dBm, the output voltage increased from 6.7 mV to 0.39 V (Fig. 4d). The primary difference in performance arouse from the test setup: the rectifier was directly driven by a signal generator, whereas the rectenna relied on harvested RF energy from a transmission antenna.

To assess energy harvesting under realistic far-field conditions, the rectenna was tested at various distances (10 cm, 20 cm, and 30 cm) from a high-frequency pyramidal horn antenna operating at low input powers (-10 dBm and 0 dBm) (Fig. 4e). Results demonstrated that at -10 dBm and a 10 cm distance, the rectenna produced an output voltage of 27 mV, which dropped to 14 mV at 30 cm. At 0 dBm, the output voltage reached approximately 0.17 V at 10 cm, falling to 20 mV at 30 cm. Beyond this distance, the rectenna could no generate usable output. These align with the theoretical expectations, where output voltage decreases with distance and increases with input power. While the device successfully demonstrates the feasibility of integrating a rectifier and antenna on to a textile substrate using scalable and low temperature fabrication methods, certain performance limitations were observed. In particular, the output voltage obtained experimentally under low input power conditions was significantly lower than the predicted by simulations (Fig. 4a vs. d). This discrepancy is largely attributed to material losses in the microstrip lines and the higher resistivity of the printed conductive inks compared to bulk metals. Additionally, the direct ink-based attachment of surface-mounted components, although effective in preserving mechanical flexibility, may introduce additional contact resistance. These factors collectively contribute to the reduced RF-to-DC conversion efficiency observed in the integrated rectenna. Nonetheless, these findings establish a functional baseline for fully printed, textile-based rectennas and underscore the importance of further optimizing ink conductivity, component attachment methods, and printed transmission line performance to enhance future device efficiency and stability.

These performance discrepancies highlight the limitations of the materials used in the rectenna’s construction. Conductive inks, while enabling low-cost, scalable and flexible printing of rectennas, introduce several performance limitations due to their material properties. As they are composed of conductive particles (e.g., silver) suspended in a polymer binder, they have higher resistivity than bulk conductors, leading to significant resistive (ohmic) losses as heat. Surface roughness, inhomogeneous particle dispersion, and multiple conductive domains with incomplete particle contact and contact resistance at junctions further elevate resistive losses. In addition, phenomena such as the skin effect and eddy currents can potentially exacerbate losses, particularly in RF applications. Moreover, localized heating can alter the ink’s properties, increasing resistance and heat generation. Over time, degradation induced by oxidation, cracking, and material wear decrease conductivity^43–47^.

The stability and reliability of rectennas also depend on strong connections between flexible substrates and RF components, which is challenging due to mechanical, thermal, and surface property mismatches. The soldering of small RF components on flexible substrates is prone to mechanical failure from folding, stretching, or other stresses^48^. The performance gap between experimental results and simulations also highlight limitations in commonly used software tools like CST Studio Suite and ADS. In addition to the characterized conductor and dielectric losses, performance degradation may also result from contact resistance at the ink–component interface, parasitic effects from printed geometries, and variability in ink deposition. While not explicitly modeled in ADS, these effects contribute to the discrepancy observed between simulated and experimental output voltages and are common in printed electronics on textile substrates. These tools cannot fully account for the unique properties of textiles, such as porosity, roughness and flexibility. Moreover, the databases for conductive inks often lack accurate descriptions of their performance compared to bulk materials.

Despite the challenges, this study demonstrated the feasibility of a fully printed, flexible textile rectenna for RF energy harvesting in the WiFi band. Using scalable, low-cost printing techniques, the rectenna was fabricated directly onto textile material, offering a promising pathway for wearable and IoT applications. However, several limitations inherent to conductive inks and textile substrates were identified: particularly related to material losses, substrate-induced performance variability, and mechanical stability.

To address these challenges, we focused on empirical evaluation of critical parameters such as ink adhesion, substrate porosity, and dielectric variability, using comparative fabrication and performance analysis across different ink formulations and substrate treatments. While theoretical models were not used directly in the manuscript, our interpretation was grounded in established relationships from antenna and microwave engineering such as the inverse relationship between dielectric constant and resonant frequency, and the impact of conductivity on RF losses. Given the applied nature of the study, we prioritized direct experimental measurements (e.g., S-parameters, output voltage, electrical conductivity) over predictive modelling.

Prior work on similar printed textile RF structures^17^ demonstrated mechanical robustness under bending, validating the approach used here. In this study, material flexibility and curing behaviour were also assessed, particularly the thermal degradation of ink **a** at elevated temperatures, which reinforced the importance of matching ink formulation to process conditions.

Future investigations should explore moisture sensitivity, long-term mechanical durability, and continued optimization of ink–substrate interactions. These findings highlight the need for refining fabrication processes and material formulations to further enhance the efficiency, repeatability, and real-world viability of printed textile rectennas for sustainable energy harvesting.

## Conclusion

This study focused on analyzing the key limitations of printed textile rectennas that hinder efficient RF energy harvesting. A flexible textile rectenna was fabricated using a scalable, low-cost doctor blade and screen-printing process, demonstrating an alternative to conventional methods that rely on rigid substrates, etching, or photolithography. The rectenna was fully integrated into textiles, specifically using Sintex 3D097 fabric with a dielectric constant of 1.22, and was designed to operate at 2.45 GHz within the WiFi band. However, experimental findings revealed that energy conversion was significantly impacted by the inherent electrical losses in conductive inks, substrate-related impedance mismatches, and mechanical deformations. These insights reinforce the need for further research into improving ink conductivity, mitigating frequency detuning effects, and optimizing rectenna design for real-world applications. Moreover, due to the limitations of current modeling tools in capturing the textile-specific characteristics such as porosity and mechanical compliance, this study prioritized experimental measurements over predictive modeling. Future work could benefit from tailored models that incorporate these textile-specific factors. By identifying these critical issues, this work lays the foundation for future advancements in textile-based RF energy harvesting, ultimately supporting the evolution of self-powered IoT and wearable technologies.

### Methods

#### Materials

The materials used in the study included Sintex 3D097 textile, with a thickness of 2.6 mm and a dielectric constant (ɛ_r_) of 1.22, silver nanoparticles (silver powder, APS 1–3 micron, 99.9%), water-based adhesive (Hydra Transfoil Adhesive E), nonconductive fluorelastomer (DAL-EL LT 302), and Clevios™ PH 1000 PEDOT: PSS.

#### Dielectric substrate Preparation

The dielectric textiles were laser-cut using a Beambox laser cutter, following the dimensions derived from simulations in CST Studio Suite. Molds of the intended antenna designs were also laser-cut on release paper for further fabrication steps.

#### Conductive ink a Preparation

Silver nanoparticles were mixed with non-conductive fluoroelastomer and acetone in a 5:3.5:1 ratio to achieve optimal viscosity and conductivity. The mixture was prepared in a flask and agitated overnight using a roller agitator.

#### Conductive ink b Preparation

Silver nanoparticles were combined with PEDOT: PSS and water-based adhesive in a 2:1:1 ratio. The solution was mixed in a flask with a magnetic stir bar and stirred overnight using a magnetic stirrer.

#### Conductive ink c Preparation

Silver nanoparticles were blended with deionized water and water-based adhesive in a 5:3.5:1 ratio. The mixture was prepared in a flask with a magnetic stir bar and mixed overnight on a magnetic stirrer.

#### Antenna and rectifier fabrication

A water-based adhesive layer was screen-printed onto the textile substrate to create a planarized surface. After drying for 30 min at room temperature, conductive ink was deposited using the doctor blade technique. The same method was applied to fabricate the antenna’s patch, ground plane, and the circuit lines and ground plane for the rectifier.

A 50 Ω SMA connector was fixed in the designed position using conductive ink, ensuring proper attachment. For the rectifier, surface-mounted (SMD) components were directly bonded to the textile substrate using conductive ink **a**. The ink’s high viscosity and strong adhesion enabled reliable mechanical and electrical connections without thermal soldering, which is incompatible with textiles. Printed pads were designed with enlarged contact areas to accommodate the ink-based connection and maintain mechanical flexibility. Vias were created by needle perforation at the designated points, with copper wires inserted to establish electrical contact between the ground plane and circuit lines.

#### Electrical measurements

Electrical parameters were analyzed using the Keysight B1500A Semiconductor Device Analyzer.

#### Measurement of reflection coefficient (S_11_) signal

A Keysight N5242A PNA-X Microwave Network Analyzer was used to measure the reflection coefficient (S_11_) of the antennas. Calibration was performed with a Keysight N7555A Electronic Calibration Module (ECal).

#### Measurement of electromagnetic losses

The electromagnetic losses (measured by the S_21_ transmission coefficient) were measured using the same network analyzer and calibration module for the reflection coefficient measurements. The signal difference between transmission and reception through a 50 Ω microstrip line was recorded.

#### Electromagnetic simulations

The microstrip patch antenna and 50 Ω transmission lines were designed and simulated using CST Studio Suite 2021, a 3D full-wave electromagnetic simulation tool, to optimize geometries and evaluate RF performance. The antenna was designed to operate at 2.4 GHz, and its dimensions were initially estimated using the standard theoretical expression:

$$\:{f}_{0}=\frac{c}{2L\sqrt{{\varepsilon\:}_{eff}}}$$

where $\:{f}_{0}$ is the resonant frequency, $\:c$ is the speed of light in free space, $\:L$ is the effective length of the patch, and $\:{\varepsilon\:}_{eff}$ is the effective dielectric constant of the substrate^34^. For the textile substrate (Sintex 3D097, $\:{\varepsilon\:}_{r}$≈ 1.7), this equation was used to determine the initial length required to achieve resonance at 2.4 GHz. CST simulations were then used to fine-tune the dimensions, validate the resonance behavior, and account for substrate thickness, surface roughness, and conductive ink properties.

The 50 Ω microstrip transmission lines were also designed in CST, with trace widths adapted for each substrate based on its permittivity and thickness^34^. These simulations helped ensure accurate impedance matching and consistent electromagnetic behavior across all printed inks (ink **a**, **b** and **c**) with specific electrical conductivities and substrate combinations. While full analytical modeling was not the focus of this work, these theoretical relationships were essential in guiding the antenna and circuit layout and in interpreting experimental results.

#### Advanced design system (ADS) simulations

The rectifier circuit was designed and simulated using Advanced Design System (ADS) by Keysight Technologies. This software specializes in RF and high-frequency circuit design, offering robust tools for optimizing rectifier circuits used in RF energy harvesting systems. The rectifier circuit was designed and simulated in Advanced Design System (ADS 2022) using a single-shunt Schottky diode topology. The simulation included a $\:\frac{\lambda\:}{4}$ matching line, input stub, DC-blocking and smoothing capacitors. The diode (SMS7630) was modeled using its nonlinear SPICE-equivalent model, including parasitic capacitance and series resistance, to enable realistic simulation of RF-to-DC conversion efficiency.

## Data Availability

Data is provided within the manuscript.
